# Home Camera-Based Fall Detection System for the Elderly

**DOI:** 10.3390/s17122864

**Published:** 2017-12-09

**Authors:** Koldo de Miguel, Alberto Brunete, Miguel Hernando, Ernesto Gambao

**Affiliations:** Centre for Automation and Robotics (CAR UPM-CSIC), Universidad Politécnica de Madrid, Madrid, Spain; koldodemiguel@gmail.com (K.d.M.); miguel.hernando@upm.es (M.H.); ernesto.gambao@upm.es (E.G.)

**Keywords:** fall detection, camera-based, elderly, home automation

## Abstract

Falls are the leading cause of injury and death in elderly individuals. Unfortunately, fall detectors are typically based on wearable devices, and the elderly often forget to wear them. In addition, fall detectors based on artificial vision are not yet available on the market. In this paper, we present a new low-cost fall detector for smart homes based on artificial vision algorithms. Our detector combines several algorithms (background subtraction, Kalman filtering and optical flow) as input to a machine learning algorithm with high detection accuracy. Tests conducted on over 50 different fall videos have shown a detection ratio of greater than 96%.

## 1. Introduction

The risk of falling is one of the most prevalent problems faced by elderly individuals. A study published by the World Health Organization [1] estimates that between 28% and 35% of people over 65 years old suffer at least one fall each year, and this figure increases to 42% for people over 70 years old. According to the World Health Organization, falls represent greater than 50% of elderly hospitalizations and approximately 40% of the non-natural mortalities for this segment of the population. Falls are a significant source of mortality for elderly individuals in developed countries.

Falls are particularly dangerous for people that live alone because a significant amount of time can pass before they receive assistance. Approximately one third of the elderly (those over than 65 years old) in Europe live alone [2], and the elderly population is expected to increase significantly over the next twenty years.

Several technologies have been developed for fall detection; however, they largely require the elderly to wear sensor devices. Some elders, especially those with dementia, tend to forget to wear such devices. Elderly individuals with dementia require special care to maintain independent living conditions. People suffering from dementia generally desire to live in their own homes; however, this is not always possible. Thirteen percent of the world’s population over 60 years old have dependent living arrangements [3]. There are approximately 7 million dementia patients in Europe alone, and this number is projected to nearly double every 20 years [4].

The use of intelligent systems in elderly patients’ homes (creating smart homes) improves their independence, comfort and safety [5] and prevents depression [6]. In addition, it frees caregivers from certain daily care tasks. In the study presented in [5], caregivers believe that these technological advances can be very useful if used conveniently, for example in areas like security (old people feel more secure ) and leisure (old people do not need caregivers to be entertained). Simply knowing that their patient is safely at home gives caregivers important psychological respite. Smart homes will allow people to extend their independent living years and reduce the time required for caregivers to monitor their elders [5]. Fall detection systems such as the one described in this paper are an important step towards smart home development.

The fall detection system proposed in this paper is based on a low-cost device comprising an embedded computer and camera. This device can be installed into walls or ceilings and monitor a room without human intervention. Furthermore, the people monitored at home are not required to wear devices. Thus, the system is capable of 24 h monitoring. It is important to indicate that this system is intended for people living alone at home because, if there is more than one person at home, and one of them falls down, the other can call for help.

The system is based on artificial vision algorithms that monitor the presence of people in a room and detect if a person has fallen. When a fall is detected, an alarm message is sent to the caregiver along with a picture. If the person recovers, another message is sent. No other privacy information is exchanged.

The main contribution of this paper is to demonstrate that a real-time fall-detection system based on vision algorithms can be executed in a low-cost device like a Raspberry Pi, obtaining good performance values (i.e., sensitivity of 96%), comparable to other systems using more expensive and more powerful hardware.

This article is structured as follows: the state of the art in fall detection is discussed in Section 2, both from the point of view of commercial technologies and advances in related research technologies. Section 2.3 focuses on computer vision techniques and describes the common concepts and procedures used for fall detection. Section 3 covers hardware, and Section 4 presents the developed algorithms for the fall detection system. Section 4.7 describes how the alert system functions. Section 5 outlines how the system is installed in a home. Finally, in Section 6, the results obtained by the present study are discussed. Section 7 concludes the paper and draws conclusions.

## 2. State of the Art

The state of the art in fall detection technologies can be divided into three categories per Mubashir et al. [7]: wearable sensors, ambient sensors and vision based technologies.

### 2.1. Wearable Sensors

The most common technologies found in these types of sensors are accelerometers and gyroscopes. These are devices that are easy to wear, but have some drawbacks as the power consumption (limiting its usability) and the sensitivity to body movement (which may cause false alarms). In addition, a considerable amount of these devices rely on a user’s ability to manually activate an alarm after a fall event. Furthermore, even if they incorporate automatic fall detection technology, these types of devices generally have a lot of false positives, based on the author’s experience.

Nevertheless, from a commercial point of view, wearable sensor technology is the most commonly utilized type of commercial device, typically taking the form of a pendant, belt, or watch. Furthermore, in research, it is possible to find several interesting approaches. Bagala et al. [8] presented 13 algorithms based exclusively on accelerometers and reported an average detection rate of 83% and a fall detection rate of 98% for the highest performing algorithm. The chief problem with accelerometer detectors lies in discriminating real falls from abrupt movements, which can generate false fall warnings. To solve this problem, Wang et al. [9] propose placing an accelerometer inside the wearer’s head. Lindemann et al. [10] propose a similar solution inside the wearer’s ear.

More advanced wearable devices incorporate multiple sensor technologies. The system presented by Mathie et al. [11] uses a single waist-mounted system of gyroscopes and accelerometers to acquire data about the inclination and movement of a subject. The interesting approach of Bianchi et al. [12] adds barometric sensors capable of sensing height variations caused by falls. They report a success rate of approximately 71%.

One of the main advantages of wearable sensors is their capacity for biometric sensors, which have great potential not only in rehabilitation, but also for fall detection. Ghasemzadeh et al. [13] present an array of sensors that can read a patient’s posture and simultaneously obtain muscular activity readings using electromyographic (EMG) sensors with a fall detection rate of 98%.

The development of mobile phone technologies, and the sensors incorporated by them, implies a very interesting option for fall detection solutions away from home. Abbate et al. [14] report a 100% fall detection rate using an algorithm based on accelerometers commonly found in mobile phones. They trained their algorithms to discard false positives generated by several common activities and achieved 100% specificity. Android’s official application store currently offers applications with this functionality; however, these applications give little to no information about their reliability.

A combination of wearable sensors and mobile phones is considered by [15,16]. The former proposes a human fall monitoring system consisting of a highly portable sensor unit including a triaxis accelerometer, a triaxis gyroscope, and a triaxis magnetometer, and a mobile phone for data processing, fall detection and messaging. In [16], mobile phones and previously validated, dedicated accelerometers are used not only to detect a fall but also to automatically classify the fall type.

### 2.2. Ambient Devices

Ambient devices measure the environment of a subject under protection. The most common technology used in this group is infra-red sensing, but additional technologies based on sound and vibration sensing are the subject of promising developments. One of the drawbacks of these systems is that they have to be installed in several rooms to cover the whole area of actuation.

Although ambient-based technologies are also used in commercial fall detection devices, they typically consist of sensors (presence, force, pressure) associated with wearable sensors and focus their detection capabilities on monitoring unusual behaviour such as subjects who do not return to bed after waking up at night [17,18,19].

Zhuang et al. [20] present a fall detection system based on audio sampling. They acknowledge that their system exhibits high detection failure rates, which they were able to decrease using machine learning algorithms. Khan et al. [21] sample environmental noise to better discern the noise made by a subject.

Another interesting approach is the use of vibration sensors embedded into flooring. Alwan et al. [22] report a 100% fall detection ratio, with the ability to distinguish activities through vibrations. Rimminen et al. [23] present another approach using electromagnetic sensors in floor plates that can create an image of objects touching the floor. They report a 91% detection success rate. However, both solutions use floor plates, similar to those presented by the “Future Care Floor” project [24], requiring large modifications to the installation environment.

The system described in [25] uses a laser based system to detect that a person has fallen. It consists of using a laser-emitting component in conjunction with a light-sensing component to generate a grid of theoretical intersections against which blocking objects are detected via instantiated intersections.

An infrared ceiling sensor network system is proposed by [26] to know the existence/non-existence of persons under the sensors, and consequently detect falls if the person remains too long in the same position.

To conclude this section, it is interesting to mention a system that activates an airbag when a fall in detected [27]. This wearable airbag incorporates a fall-detection system that uses both acceleration and angular velocity signals to trigger inflation of the airbag.

### 2.3. Vision-Based Devices

Most commercial fall-detection systems in the market are based on portable devices, as shown in [28]. Nowadays, it is not easy to find commercial devices using computer vision, but their associated technical advancements and related literature remain promising.

Vision-based devices have the same drawback as ambient devices: they have to be installed in several rooms to cover the whole area of actuation. Another drawback is the treatment of privacy: how to deal with images from a real person’s life. For these reasons, streaming is controversial. In our case, the systems sends only images when the fall has been detected. These images can be blurred easily to avoid facial recognition from third parties.

Some advantages of these systems are that they can run is many computers, and that there are many algorithms and libraries implemented open-source. Although a variety of algorithms have been developed for fall detection, some of which are designed to analyse static images or treat each frame individually, a number of characteristic steps are frequently found in most fall detection systems, which are explained in the following subsections.

Some systems also merge cameras (Microsoft’s Kinect) and accelerometera, like the one use by [29], where a fuzzy system merges sensor data to determine if a fall has occurred.

#### 2.3.1. Cameras

In vision-based systems, cameras are one of the most important parts. Following the discussion presented in [7], the vision based approaches are focussed on the real-time execution of the algorithm using standard computing platforms and low cost cameras. There are several methods used to obtain the semantic information through the video analysis. Many of them made use of a 2D or 3D model, and others are based on the extraction of some features after the video image segmentation of the body. A more detailed explanation of those approaches could be found in [7] where they are classified into the following categories: body and shape change, posture detection, inactivity, spatiotemporal and 3D head change.

In addition, two types of cameras are mainly used for fall detection: 2D cameras (like the one used in this paper or in [30], and 3D time of flight (ToF) cameras like in [31,32]. The lateral resolution of time-of-flight cameras is generally low compared to standard 2D video cameras and are much more expensive.

#### 2.3.2. Processing Units

It is important to remark that most vision-based fall detectors need a high computational processing power or expensive hardware (cameras or computers). An active vision system for the automatic detection of falls and the recognition of several postures for elderly homecare applications is presented in [31]. It uses the TOF (Time of Flight) MESA SwissRanger SR3000 (Zurich, Switzerland), which is quite expensive.

A scheme based on foreground extraction, feature extraction and intelligent fall detection, using a Intel^®^ Core™ i5 processor (Santa Clara, CA, USA) at 2.6 GHz is used by [30]. A novel approach in fall detection by performing an analysis on Riemannian manifolds, detecting falls from a single camera with arbitrary view angles is presented in [33]. Tests have been done on a computer with an Intel^®^ Core™ i7-2600 3.40 GHz processor, and 16 GB of RAM clocked at 1333 MHz.

A heterogeneous platform Zynq-7000 SoC (System on chip) platform is used by [34], which combines an ARM Cortex™ A9 processor and a FPGA (Field-programmable gate array), producing a very fast low power device.

As we see, most of the vision-based fall detection systems use powerful and expensive hardware. The system presented on this paper works with a Raspberry Pi, a very cheap board that will be described in Section 3.

#### 2.3.3. Relevant Information Segmentation

The segmentation of relevant and background scene information is a common first step for many computer vision algorithms. The most basic techniques involve the subtraction of a background image or the registration of scene changes from one frame to another. More advanced algorithms, such as the background subtraction algorithm developed by Zivkovic [35], register the most common colours of each pixel and update learned data over time, consequently exhibiting adaptive capabilities responsive to scene changes over time.

The recognition of specific features can also be used to extract relevant information from a scene. Feature descriptor algorithms, such as histograms of oriented gradients, can be trained to identify certain features of the human body. One example of this application can be found in Rougier et al. [36,37], where a subject’s head and body are independently followed, and accurate readings of their relative trajectories over time are found.

#### 2.3.4. Subject Tracking

The tracking of a subject’s evolution from one frame to the next is another typical step in fall detection algorithms. This requires either identifying a recognizable feature of the subject to be followed or using previously obtained data to estimate the new position of the subject, which can be achieved with Kalman filters or recursive Bayesian filters. Regarding feature tracking, Yilmaz et al. [38] propose using colour and texture to track a subject. Yakhu and Nikom [39] propose a simplified colour based algorithm using the assumption that the colour of a person’s head, body and legs are easily differentiable.

#### 2.3.5. Data Modelling

To acquire useful information for fall detection, a subject’s acquired data must be modelled. One of the most common modelling methods used is the approximation of geometric shapes, a cost effective method for data modelling with reliable results that can be combined with other techniques to analyse complex situations. The most conventional approaches use rectangles and ellipses [40,41], which allow for easy sampling of data such as height, width and subject angle.

A more advanced approach is taken by Yao and Odobez [42], who have developed a multi-camera system to register three-dimensional subject data, modelling them as three cylinders for increased accuracy.

#### 2.3.6. State Classification

A decision system is typically required in a fall detection system. In wearable systems, rules and data thresholds are customarily used, which have also been applied to computer vision fall detection [43,44], but are usually seen as limited for this scope of detection.

Machine learning techniques have been widely applied to computer vision fall detection since 2006 [45]. Data classification algorithms such as K-Nearest Neighbours [46] and Support Vector Machines [47] have been successfully used in computer vision fall detection, and more advanced machine learning algorithms such as Artificial Neural Networks [48] and Hidden Markov Models [41] have also been applied, generally with great success.

## 3. Description of the Hardware

Our detection system (called “Fallert”) was originally developed to be executed on a low-cost embedded computer. Several options were taken into account, and the Raspberry Pi 2 board was chosen due to its sound technical characteristics, widespread adoption and low price.

In addition to the board, the camera module designed for Raspberry Pi was used, which uses the CSI (Camera Serial Interface ) port of the board and thus significantly fewer CPU (Central Processing Unit) resources than a regular USB camera.

A few additional items are required for each detection unit: cases for both the embedded computer and camera, an SD card that acts as main memory for the embedded computer, a lens that enlarges the angular range of the camera module, a WiFi adapter and a power supply unit for the board.

**Figure 1:**
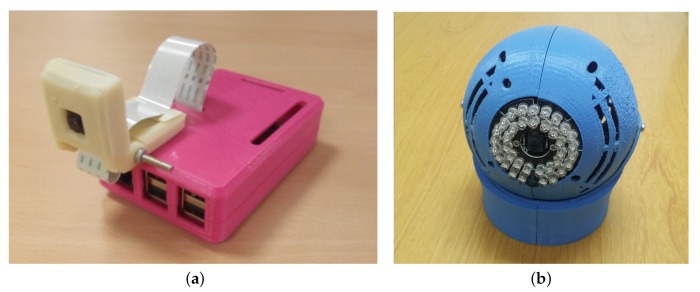
Fall detection system prototypes. (**a**) first prototype; (**b**) second prototype.

The initial prototype (Figure 1a) is a fully capable independent fall detection system with an estimated cost of 91€. As mentioned above, this unit is based on a Raspberry Pi 2. Currently, a new version of the unit is being developed based on the Raspberry Pi 3 board (Figure 1b), which includes WiFi connection and consequently the price will be reduced.

As it was said before, there are not many vision-based commercial devices for fall detection nowadays. In fact, the top 10 fall detectors in [28] are based on portable devices.

Regarding vision-based system, it is possible to find [49], an online system similar to the one presented in this paper, based on IP cameras, but where the fall detection algorithms run outside the camera in a server. As it was explained before, the research projects on fall detection based on cameras use powerful computers or cameras.

## 4. Fall Detection Algorithm

The objective of our algorithm is to distinguish subjects in a fall state. To achieve this goal, the algorithm extracts data from the subject in a scene to recognise his/her current state.

Data acquisition requires multiple preliminary steps: subtracting the subject from the background, progressively learning the subject’s changing environment and identifying uninteresting objects (to facilitate their rapid recognition as background), following the subject through the scene and identifying subjects that are partially occluded by furniture.

A Kalman filter is used to reduce noisy data and absorb the repetitive periodic changes common to various human actions. Finally, a machine learning system is applied to the acquired data to classify the subject’s current state.

**Figure 2:**
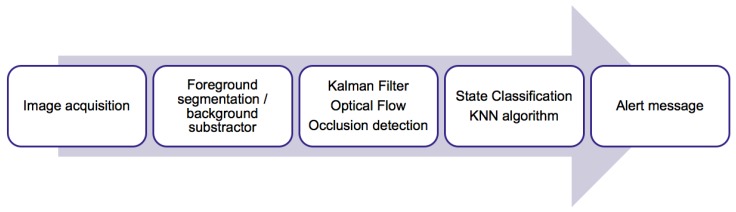
Model diagram.

A model diagram of the system is shown in Figure 2 and it is explained in the following subsections. It has been developed in C/C++ using the OpenCV library, a popular and well supported computer vision library.

### 4.1. Data Model

The data model is based on three variables: the angle, ratio and ratio derivative. The angle refers to the angle between the *x*-axis and the major-axis of an ellipse enclosing the subject. The ratio refers to the relationship between the width and height of a rectangle that encloses the subject. The derivative value of the absolute normalized ratio variation measures how quickly the perceived silhouette changes over time. All three parameters can distinguish people in situations such as walking, standing, sitting, falling, etc. The chosen parameters for fall detection are independent of the subject’s distance to the camera; however, due to limited camera resolution, distances over 10 m are undesirable in our case.

In addition to the aforementioned parameters, other variables must be registered for proper operation of the developed algorithms, including the area, the dot cloud generated by optical flow (Section 4.4) and the rectangle and ellipse that accommodate the subjects in a scene.

**Figure 3:**
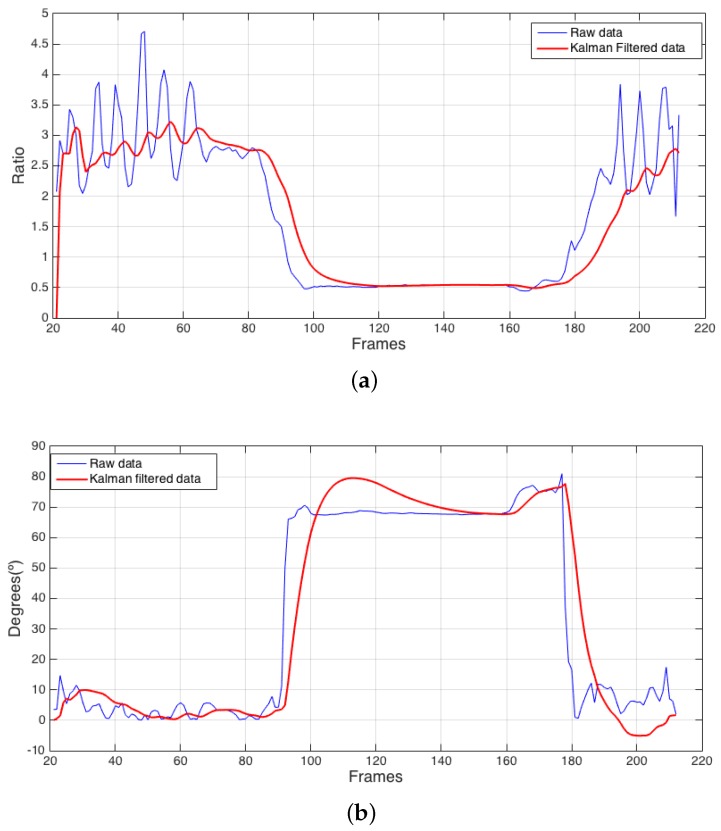

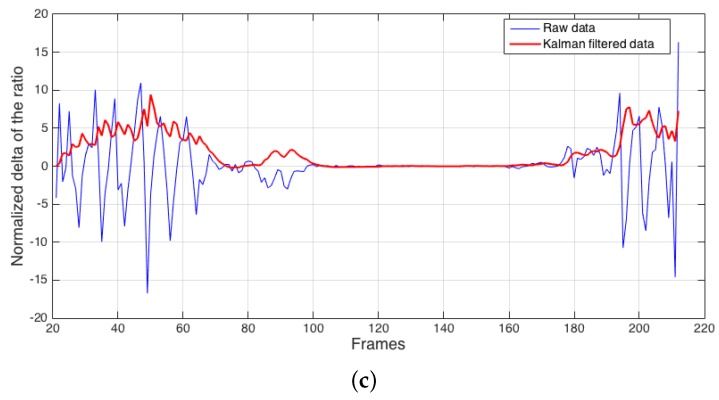
Example of a fall occurring perpendicular to the camera. (**a**) ratio; (**b**) angle; (**c**) normalized delta of the ratio.

**Figure 4:**
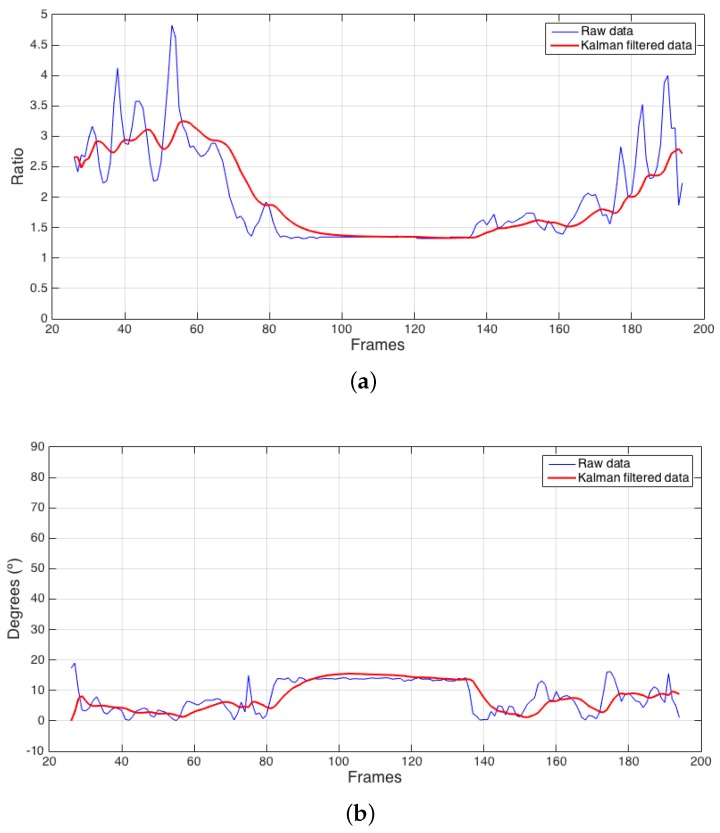

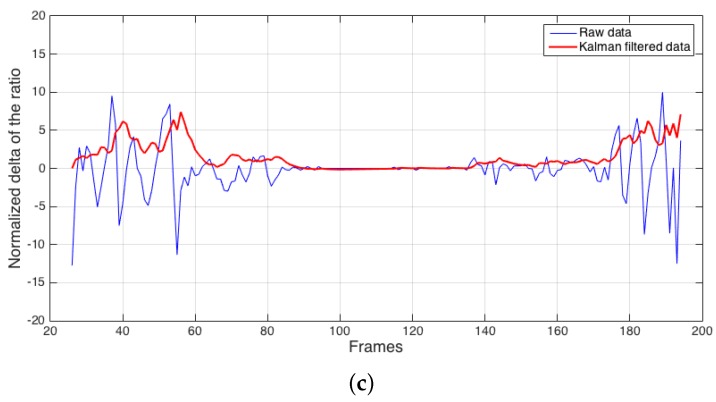
Example of fall occurring parallel to the camera. (**a**) ratio; (**b**) angle; (**c**) normalized delta of the ratio.

Figure 3 provides data corresponding to a fall oriented perpendicular to the camera, and Figure 4 shows data corresponding to a fall oriented parallel to the camera.

Figure 3a and Figure 4a represent the subject’s ratio, and Figure 3b and Figure 4b represent the subject’s angle relative to what the camera perceives as perpendicular. In all the subfigures, blue lines represent the data obtained by directly modelling the subject, and red lines represent the same variables after Kalman filtering (Section 4.3). In Figure 3c and Figure 4c, the blue line represents the subject’s normalized ratio variation from one frame to the next, and the red line represents the result of applying Kalman filtering to the absolute value of this variable, which is used to measure how quickly the subject’s contour changes over time.

The graphs in Figure 3 and Figure 4 show the subject entering the scene at the beginning of the timeline. During the first portion of the timeline, the subject walks through the room. As the fall begins to occur, both examples indicate a transitional period where the subject progresses from his regular walking state to a stable fall state. A similar transition is observed when the subject recovers from the fall. The central area with stable values corresponds to the stable fall state. During the last stage of both examples, the subject stands up after the fall and exits the scene. Falls at other angles relative to the camera present different characteristic values for these variables.

The algorithm’s stable fall criteria depend on the data used to train the machine learning algorithms (Section 4.5). Currently, the training data for the stable fall state includes a subject who is: (1) unconscious after a fall and (2) conscious but unable to travel.

### 4.2. Background Subtractor

The background substractor algorithm is used to remove the background and obtain the foreground contour of the person in the scene. The background substractor that we use in the system described in this paper is aimed at daylight situations. For night situations, we consider to use a different algorithm, as explained in the future work section.

**Figure 5:**
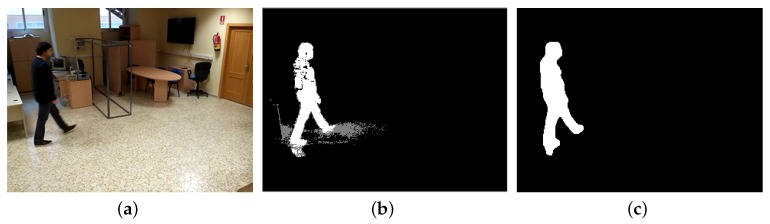
Foreground extraction. (**a**) original image; (**b**) extracted foreground. Areas detected as shadows are coloured in grey; (**c**) final cleaned foreground mask.

The “backgroundSubtractorMoG2” algorithm, which is named after the mixture of Gaussians technique it applies, developed by Zivkovic [35] is used for background subtraction. The background SubtractorMoG2 algorithm is a colour based algorithm that learns a set of frequently seen Gaussian pixel components and updates learned data over time as additional images are analysed. It also features limited shadow detection (Figure 5b).

**Figure 6:**
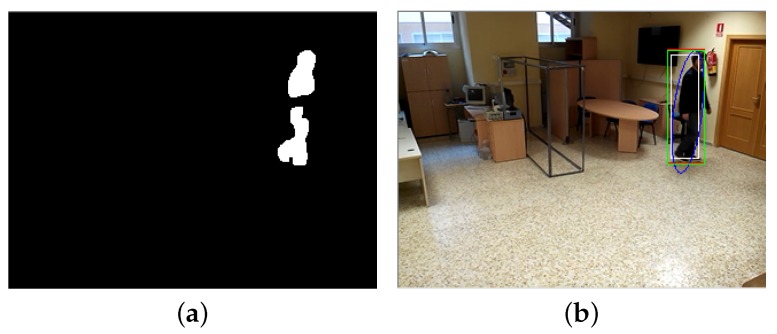
Broken contour reconstruction example. (**a**) image with separated contours; (**b**) image with reconstructed contour.

The extracted foreground is modelled as an array of contours checked against the predicted data generated by the Kalman filter for each user. Occasionally, the background subtraction system generates broken contours when a subject moves through an identically coloured object, and an aggregate contour is generated by adding the information of each individual broken contour associated with the subject (Figure 6).

Contours that exhibit interesting characteristics but do not match previous subjects are stored as potentially new subjects. The algorithm occasionally registers small changes in a scene for no longer than a few frames; however, these small changes rarely endure for longer than a few frames and exhibit motionless object characteristics detected by the optical flow algorithm; thus, they are registered as uninteresting changes in the scene.

The background subtraction algorithm implemented in OpenCV is unable to discriminate what should be learned in a scene. Thus, if a fast learning rate is applied, the algorithm will easily adapt to changes in the environment and rapidly learn about displaced objects in the scene. On the contrary, if a subject remains stationary for a few seconds, the algorithm learns that the subject is part of the scene. If slow learning is applied, these advantages and disadvantages are inverted; however, neither situation is desirable.

To solve this problem, a selective learning system has been implemented. This selective learning system uses conclusive information generated from analysing the current frame to determine the areas that should not be learned. These areas are substituted for the expected background as the ideal background. This allows for fast learning rates without running the risk of the subject being learned as part of the background.

The selective background learning system offers the following advantages: subjects in the scene are not learned as background, rapid learning of progressive changes of illumination and objects recognized as uninteresting are rapidly learned to be part of the background.

The selective learning system executes the background subtraction algorithm twice per video frame. The first execution generates a foreground information mask to analyse the frame, and the second execution performs selective learning of the scene. This makes the selective background learning system the most computationally expensive algorithm in our fall detection system.

### 4.3. Kalman Filter

A Kalman filter was chosen to keep track of the subjects in the scene. The Kalman filter was chosen instead of other algorithms like (exponential) moving average filters because its prediction properties, noise reduction properties and fast response.

Kalman filters address noisy and imprecise data and generate predictions of new states based on past data. A lineal version of the filter was implemented utilising data extracted from the subjects in a scene. The chosen parameters are differentially processed by the filter depending on whether they are used for fall detection or future state prediction. The following equations are applied:

Centre of mass *x* and *y* position:

(1) $$\begin{array}{c} x\left(k\right)=x\left(k-1\right)+\dot{x}\left(k-1\right)\cdot dT, \end{array}$$

(2) $$\begin{array}{c} y\left(k\right)=y\left(k-1\right)+\dot{y}\left(k-1\right)\cdot dT, \end{array}$$

(3) $$\begin{array}{c} \dot{x}\left(k\right)=\dot{x}\left(k-1\right), \end{array}$$

(4) $$\begin{array}{c} \dot{y}\left(k\right)=\dot{y}\left(k-1\right), \end{array}$$

Height and width:

(5) $$\begin{array}{c} h(k)=h(k-1), \end{array}$$

(6) $$\begin{array}{c} w(k)=w(k-1), \end{array}$$

Ratio change speed:

(7) $$\begin{array}{c} V_{Ratio}\left(k\right)=V_{Ratio}\left(k-1\right)+{\dot{V}}_{Ratio}\left(k-1\right)\cdot dT, \end{array}$$

(8) $$\begin{array}{c} {\dot{V}}_{Ratio}\left(k\right)={\dot{V}}_{Ratio}\left(k-1\right), \end{array}$$

Angle of the ellipse adjusting to the individual:

(9) $$\begin{array}{c} \phi \left(k\right)=\phi \left(k-1\right)+\dot{\phi }\left(k-1\right)\cdot dT, \end{array}$$

(10) $$\begin{array}{c} \dot{\phi }\left(k\right)=\dot{\phi }\left(k-1\right). \end{array}$$

The parameters used for fall state detection are ratio, ratio change speed and angle. The main functions of the filter are to reduce measurement noise and absorb periodic changes characteristic of specific movements such as walking.

**Figure 7:**
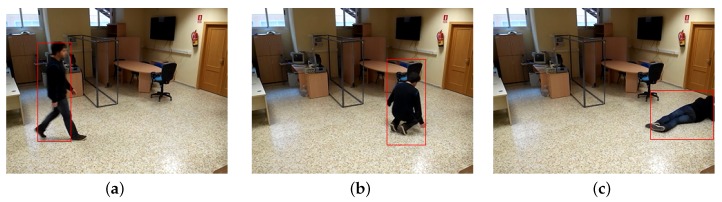
Kalman filter predictions for subject position. (**a**) Step 1: walking; (**b**) Step 2: fall initiated; (**c**) Step 3: fall terminated.

The parameters for future state prediction are the centre of mass and ratio. The centre of mass is filtered to reduce noise and acquire a quicker response to changes. Noise was found to be negligible compared with subject size and was independent of a subject’s proximity to the camera. The position prediction system is used to associate the data of each obtained frame with previously seen subjects in a scene (Figure 7).

### 4.4. Optical Flow

**Figure 8:**
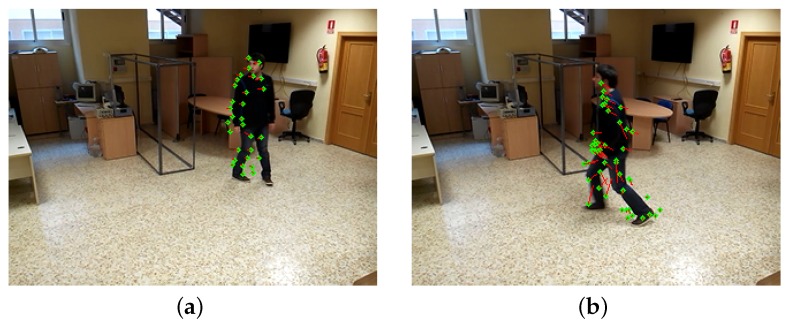
Optical flow applied to the subject in a scene. (**a**) Subject rotating. (**b**) Subject getting up after a fall event.

Optical flow is used to keep track and eliminate static objects that appear in the scene. As implemented in OpenCV, optical flow adheres to the method originally described by Lucas-Kanade [50,51]. It is employed to track a set of points in areas of interest from one frame to the next. Under the current implementation, optical flow is chiefly used to measure the movement of the elements of interest in a scene (Figure 8) and identify static objects after they have been moved, as well as other changes in a scene, which is crucial for the selective learning algorithm. The system was sensitive enough to detect even small movements typically made by people standing in fixed locations.

Although the system performs well on static object recognition, people are more challenging to track for lengthy time periods because their clothes fold, they turn around, etc. To solve this problem, a statistical method is used to recognize and remove any points associated with odd behaviour and, when required, acquire a new set of points to describe a person in a scene. This statistical method works as follows: in first place, 40% (experimentally obtained) of the points in the cloud with a movement distance closer to the average for that cloud (since the last frame) are selected as base metric. Then, the cloud points differing more than 1.5 times from the standard deviation are flagged as doubtful. Finally, any doubtful point that does not move in the same way as the rest is removed after three frames.

Generally, discarded points present a rather typical behavior caused by the element to which they were associated, which has probably disappeared by the movement of the person. These points generally present a strong random movement compared to the rest of points and are generally associated with a background element that is close to the person. Consequently, these points are isolated from the rest of the cloud of points, and, in the following frames, present no movement or independent movement to the rest of the cloud.

### 4.5. State Classification

The k-Nearest Neighbours (KNN) algorithm is used in the present system for subject state recognition. KNN is a machine learning classification algorithm, wherein each new set of data read from a subject is compared to the k most similar known data, and a value, fall or not fall, is given to each possible state depending on the state and distance to the k nearest neighbours. KNN main drawback is its execution time due to its lack of data pre-processing, making each new data that has to be classified to be checked against the “k” most similar data of the unprocessed training set. However, the performance of KNN was more than adequate for our fall detection implementation.

The KNN algorithm takes the angle, subject ratio and three most recent values for ratio change speed as input. The output is either a fall or not fall state. The value of three was experimentally chosen and it can be optimised in future work.

The input variables were normalized and weighted to reduce the influence of the otherwise triplicated ratio change speed. The rationale for using ratio speed change is twofold. First, it allows for the addition of a calmness condition to the fallen state, which was trained in the present fall detector to allow for movement ranging from calmness to small movements to seizure-like movements on the floor. Second, the inclusion of ratio change speed avoids inconsistent state transition phases.

A low “k” value would be sensitive to failing due to noisy training data, and a high “k” would increase the calculation time. Tests were performed with “k” values between one and five, and finally “k” was set to three since it presented easily negligible errors, and the computation time was acceptable.

A training dataset was generated by manually identifying the time intervals where a fall happened in the training videos. Then, the relevant variable data was extracted from the videos, associated with the correct state and added to the training data file. More information can be found in Section 6.1.

The variables in the present study were able to distinguish the most common states. Certain actions, such as sitting in certain positions or angles toward the camera, may be similar to certain falling positions from the camera’s viewpoint; however, these cases could be easily assigned separate states, allowing for specific algorithms to be applied on demand when they are detected. This allows for an intentional performance algorithm to be used for general state detection and for specific algorithms solely targeting the subjects in a specific state to be used only when necessary.

The system is open in future versions to use a different state classification algorithm such as support vector machines (SVM), which have been documented to be appropriate to study human activities [52].

### 4.6. Occlusions

Occlusions occur when a relevant area of the bottom of a person is covered. Occlusions can be found by applying a series of geometrical rules regarding a subject’s perceived shape at the moment of the occlusion versus that of the immediately preceding frames. These geometrical rules consider the subject’s perceived surface and the spatial position of his/her upper body. The system also recalls geometrical data about the subject to detect when an occlusion has ended.

Currently, only inferior occlusions are considered, when the subject moves behind an object and consequently a part of his/her body disappears. This event is detected when the lower area of the perceived contour disappears over a small period of time, while the upper area presents a continuous profile over the same period of time.

**Figure 9:**
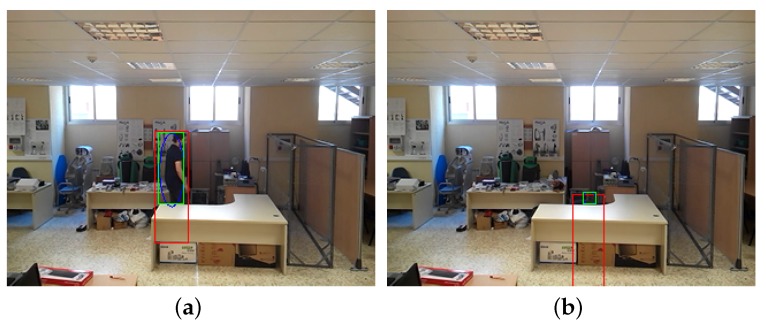
Example of an occlusion caused by a desk. (**a**) subject occluded by a desk; (**b**) an occluded fall behind a desk.

When a fall behind an obstacle causes a subject to be hidden from the camera, the machine learning fall detection algorithm is not used, and fall detection consists of searching for the subject’s disappearance under coherent conditions for the detected occlusion (Figure 9).

The height of the subject is stored at the initiation of occlusion. This allows for the regular detection regimen to be reactivated after occlusion detection is completed.

### 4.7. Alert System

For the system to be useful, it must relay fall events to specific responsible persons or entities. At this stage, a dedicated software for fall event communication was not developed in-house; rather, two existing communication solutions were utilised.

Email communication using the Mutt email client for Linux [53] and the popular messaging system Telegram, using the Linux Telegram Messenger CLI (Command Line Interface) developed by Vysehng [54], were applied. Both Mutt and Telegram are sanctioned under the GNU General Public License.

The alert system features a 2 s delay prior to sending fall alerts to avoid sending alerts under peripheral conditions where a subject is shifting from a regular state to a fall state and vice versa, which generates a brief period of time wherein the read state is not yet stable.

The 2 s delay also removes a small number of false positives currently classified as unstable states. Such false positives are a main focus area for system improvements and are further detailed in the results and annotation section (Section 6.3).

**Figure 10:**
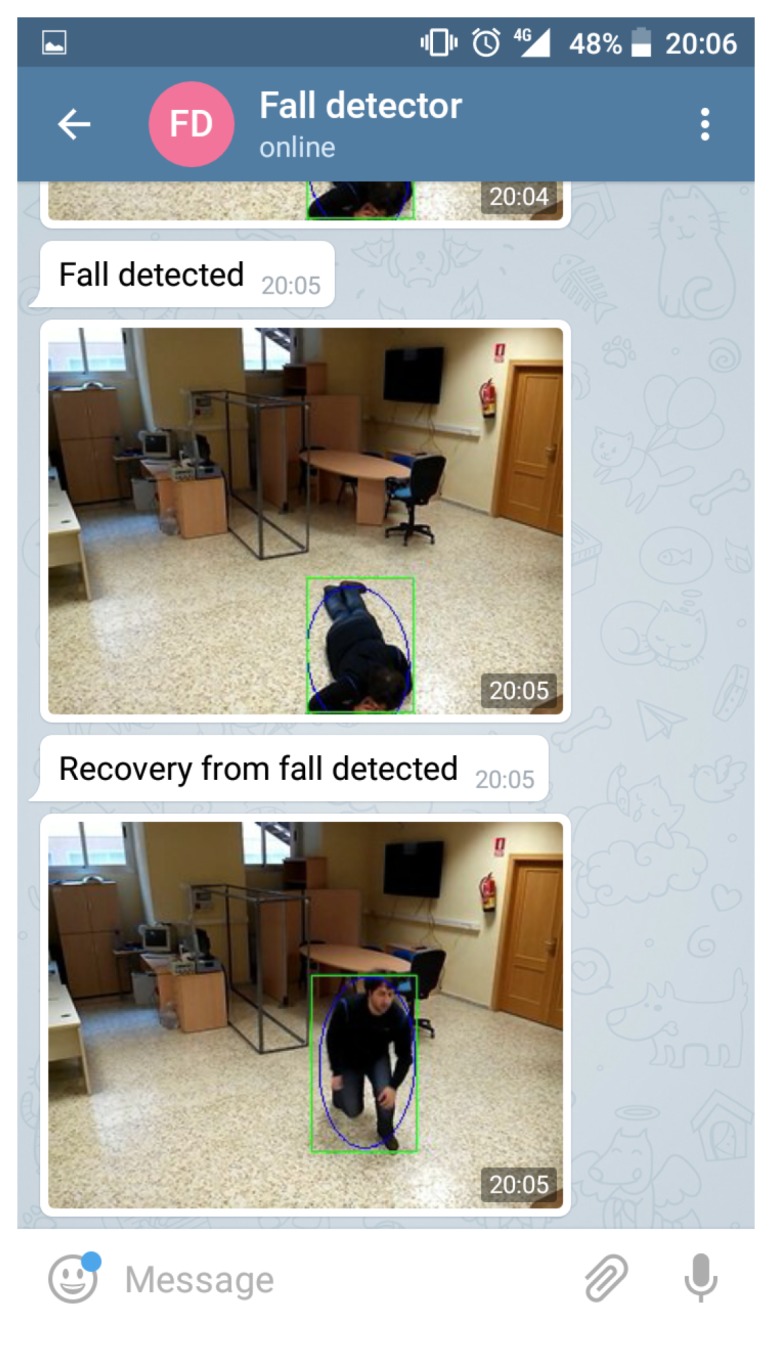
Fall warning delivery example.

The delay system also has potential for addressing when a fallen subject unsuccessfully attempts to rise, causing a recover event followed by a subsequent fall event a few seconds later. An example message is shown in Figure 10.

Although privacy in communications has not yet been taken into consideration, the system is based upon the concept of sending only subject’s information once an accident has happened, thus avoiding active monitorization and streaming of data. At this point, it is extremely relevant to mention the study of Londei et al. [55], which was financed by the Social Sciences and Humanities Research Council of Canada and studies the perception of sending images on the detection of fall events. Surprisingly, they found that the majority of the elders (92.6%) and caretakers (82.4%) questioned were in favour of using untreated images of fall events, even for locations such as bathrooms, if it results in an improved response to a fall event. However, the experts acknowledged that using filters to reduce image details to the minimum required for fall recognition would be preferable.

## 5. System Installation

The system is designed to have a camera installed at an approximate height of 2 to 2.25 m. The tilt angle used in the present study was approximately 14° ± 5°. The use of a larger angle and a wide angle lens helps to minimize the blind spot under the camera. The camera must not be oriented towards TVs or reflective objects (including floors), and the scene must not be dominated by bright windows.

**Figure 11:**
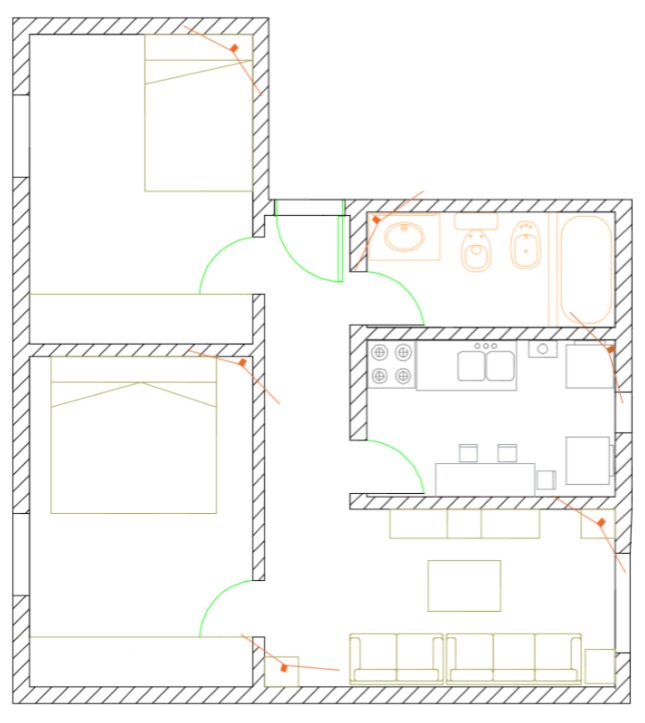
Example of system installation in a home.

Figure 11 shows a flat type commonly associated with the elderly consisting of two bedrooms, a living room, a kitchen, a bathroom and a hallway. The proposed camera positions, highlighted in orange, indicate the approximate angle using a wide angle lens. The entire flat can be covered with six cameras.

Certain elements, such as halogen lights and large windows, can induce scene wide overexposure and generate further colour information loss over the entire scene. In addition, a real home environment can generate complex scenes: tables and chairs may occlude an observed subject, or a television may become a source of continuous changes. These issues can be reduced by placing the fall-detector pointing to static scenarios.

## 6. Results and Discussion

### 6.1. Dataset

To measure the system’s performance, a total of 53 videos were recorded in two different locations: a laboratory and a house. The videos recorded in the laboratory are divided in four groups. The first group includes 24 general fall videos. These videos depict falls in diverse directions and locations within the same room. From this set, 16 videos were chosen to generate training data, and eight videos were chosen to measure detection performance. This dataset included videos with falls in the main four direction relative to the camera to make sure all cases were equally represented.

The second group includes four occlusion videos: two of these videos portray occluded falls. Because occlusion detection is not achieved via machine learning, these videos were all used for testing.

The third group includes 14 sitting videos: These videos show different locations within a room. From this set, eight videos were labelled as training data, and the remaining six were used for testing detection performance.

The fourth group includes two miscellaneous videos: These videos depict the subject in the scene minus fall, occlusion and sitting events. Both of these videos were used for testing detection performance.

In the second group (inside a house), 14 videos were recorded. These videos were obtained from a location with very different luminic conditions to those observed in the other videos. This set includes six fall events, two sitting videos, three occlusions, one occluded fall, and two miscellaneous activity videos. All the videos from this set were used for testing detection performance.

With the exception of the house video set, all the videos were shot in the same laboratory across multiple takes on different days and times. The laboratory light conditions are predominantly artificial with some natural light entering through some windows. The 14 videos recorded in the house were shot on a home environment with large amounts of natural light entering from a glass. Both places feature their regular furniture during the filming. Videos are between 20 and 50 s long.

During the shots, the subjects were instructed to move regularly through the scene and at some point execute the planned activity for that video and then leave the scene afterwards. The planned activities included falling in various directions and places in the scene, walking through the scene, being occluded behind some furniture with or without a fall, sitting and interacting with some objects.

To best utilise the first set of fall videos, a second set of eight different videos were chosen, and a new training data set was generated without changing the other videos. Because the videos for every other category obtained the same results in both tests, only these new eight fall videos were added to the results for performance analysis.

### 6.2. Parameters Used for Performance Measurement

The parameters used to evaluate the tests are sensitivity (the percentage of fall events detected), specificity (the percentage of events without falls detected correctly), precision (the percentage of fall alerts that represent actual falls) and accuracy (the percentage of correctly detected events), defined as follows:

(11) Sensitivity=TruePositives/TotalPositives,

(12) Specificity=TrueNegatives/TotalNegatives,

(13) Precision=TotalPositives/(TruePositives+FalseNegatives),

(14) Accuracy=(TruePositives+TrueNegatives)/TotalEvents.

Of all these parameters, sensitivity is most important because the main objective of a fall detector is to detect all fall events. Accuracy and precision are also fairly interesting from a detection performance point of view.

### 6.3. Results Obtained

The results of the present study are summarized in Table 1 and Table 2.

The false positive in the “walking between falls” category was generated from a video in the second set of fall videos and was caused by a carpet that folded due to the subject’s fall. After the subject recovered from the fall, the carpet began to slowly unfold, which bypassed the static object detection system and eventually generated a false positive.

The fall event that was labelled as incorrectly detected corresponded to a fall event that, although correctly detected as a fall, generated an incorrect fall recovery event while the subject remained on the floor. This was caused by the subject momentarily acquiring a position that generated data similar to a sitting position.

Similarly, although all the sitting events were correctly detected, three of them generated very brief fall detection events. However, all these fall events were correctly dismissed by the alert delay system by labelling them as unstable state transitions. The brief fall events were generated by sitting postures with data characteristics similar to certain types of falls. We are currently considering a long term solution to this issue involving the acquisition of basic but reliable posture information from the point cloud generated through optical flow (Section 4.4) when required.

### 6.4. Performance of the System

Our fall detection software currently performs at a speed of approximately 7–8 analysed images per second in Raspberry Pi 2 configured to its default CPU clock. The background substractor and selective learning algorithms are associated with greater computational cost as they process the entire 320 × 240 sized image.

The software’s performance is currently limited by the non-optimized threading of the selective learning algorithm, which makes three of the four CPU cores perform at approximately 45% load for the current embedded computer. We plan to implement threading improvements that should solve this issue by releasing a greater amount of resources from the CPU.

In order to evaluate the performance of our system, a comparison with other vision-based fall detector systems is commented in the following paragraphs. The vision-based pedestrian fall detection system with back propagation neural network developed by [30] reports, depending on the scenario, a sensitivity from 96.6 to 100%, an accuracy from 86.3 to 94.1%, and a specificity from 72.2 to 86.4%.

The active vision system for the automatic detection of falls and the recognition of several postures for elderly home-care applications presented by [33], reports a sensitivity from 98.55 to 100% and a specificity from 95.84 to 97.25%, depending on the dataset.

The low power architecture standalone fall-detection system based on computer vision designed by [34] reports a precision from 72.70 to 80%, and an accuracy from 79.60 to 85.40% in test and train datasets, respectively.

In [17], a comparison amongst several fall detector systems in presented, showing sensitivities from 71 to 100%, specificities of 73% and accuracies of 94%.

That is to say that, compared to the aforementioned systems, our performance values (sensitivity of 96%, specificity of 97.6%, precision of 96%, and accuracy of 96.9%) seem to be reasonable, considering that we are using a much more cheap and much less powerful hardware. Our goal is to demonstrate that an embedded low cost system can have as good results as other more powerful and expensive systems.

It is important to highlight that while we are using a Raspberry Pi equipped with a Cortex 900 MHz ARM Cortex™ -A7 processor, the system presented in [30] uses an Intel^®^ Core™ i5 processor (2.6 GHz) processor, the system described in [33] uses an Intel^®^ Core™ i7-2600 (3.40 GHz) processor and the system reported in [34] utilises a heterogeneous platform Zynq-7000 SoC (which combines an ARM Cortex™ -A9 processor and FPGAs, producing a very fast low power device). Considering the camera used, our system works with a RaspiCam (about 25€), while, for example, the camera in [31] is a TOF (Time of Flight) MESA SwissRanger SR3000, which is quite expensive (hundreds of euros).

Table 3 shows a comparison of the aforementioned fall detection systems.

## 7. Conclusions

Although the system presented in this paper is currently under development, it is already able to reliably detect falls in controlled environments, while taking into account several common events found in real settings. The system performs with approximately 96% efficiency in controlled environments.

Therefore, we have demonstrated that an integral low-cost fall detection system based on computer vision techniques is possible. The present system has the enormous advantage that a person under surveillance is not required to wear a device.

We have presented different algorithms for fall detection and its differentiation from other states such as walking and sitting. In essence, we combine a background subtractor, Kalman filter and optical flow as input to a machine learning decision system to identify fall occurrences. The system’s reliability has been proven with over 50 videos, and its resulting performance is consistently greater than 96%.

Future work will focus on the improvement of the algorithm in terms of occlusion, state differentiation and illumination changes. As explained before, the system described in this paper is designed for daylight situations. Although the image quality associated with low-cost cameras has improved over time, certain light and ambient conditions continue to have an extensive impact on image quality and can generate high variance over time. Furthermore, night-time coverage using cost-sensitive camera solutions exacerbates these image quality issues. We are working on using different background substractors’ algorithms depending on the time of the day or the luminosity.

On another note, the nature of an observed subject must also be considered. Elderly individuals can show great variability in their movement patterns based on their health and age. Walking aids are commonly used by the elderly, and, during a fall event, they can easily interpose between the subject and camera or confuse the detection algorithm by disturbing the perceived shape or size of a fallen subject. Optical flow is expected to take a larger role in future developments to improve fall detection accuracy as a colour independent method for tracking subjects through a scene and acquiring new data.

## Figures and Tables

**Table 1:** Fall detection results.

Event	No. of Events	True Positives	False Positives	True Negatives
Falls	22	21	0	-
Occluded falls	3	3	0	-
Total positive events	25			
Sitting	10	-	0	10
Occlusions	5	-	0	5
Walking between falls	22	-	1	21
Miscellaneous	4	-	0	4
Total negative events	41			
Total events	66	24	1	40

**Table 2:** Algorithm performance.

Parameter	Result
Sensitivity	96%
Specificity	97.6%
Precision	96%
Accuracy	96.9%

**Table 3:** Fall detection system comparison.

System	Sensitivity (%)	Accuracy (%)	Specificity (%)	Estimated Cost	Cpu
[30]	96.6–100	86.3–94.1	72.2–86.4	900	Intel^®^ Core™ i5 2.6 GHz
[33]	98.55–100		95.84–97.25	1300	Intel^®^ Core™ i7 3.4 GHz
[34]		79.6–85.4		∼400	Cortex™ -A9 + FPGAs
[17]	71–100	94	73		Several
Fallert	96	97.6	96.9	91	Cortex™ -A7 900 MHz

## Abbreviations

The following abbreviations are used in this manuscript:

CLI	Command Line Interface
CPU	Central Processing Unit
CSI	Camera Serial Interface
EMG	Electromyographic
KNN	K-Nearest Neighbours
